# Comparative Outcomes of Mini-Percutaneous Nephrolithotomy and Standard Percutaneous Nephrolithotomy for Renal Stones: A Prospective Comparative Study

**DOI:** 10.7759/cureus.110703

**Published:** 2026-06-11

**Authors:** Md. Asaduzzaman Asad, Mohammed Monowar-Ul-Haque, Mofizur Rahman, Md. Mostafizur Rahman, Shiba Prasad Nandy, Sumaya Tasnim Maria, Hasmot Ali Mia, Md. Mahmudul Hasan

**Affiliations:** 1 Department of Urology, Kaunia Upazila Health Complex, Rangpur, BGD; 2 Department of Urology, Chittagong Medical College Hospital, Chattogram, BGD; 3 Department of Urology, Medical College for Women and Hospital, Dhaka, BGD; 4 Department of Urology, Jamalpur Medical College, Jamalpur, BGD; 5 Department of Epidemiology, Centre for Medical Research and Development, Dhaka, BGD; 6 Department of Urology, Rangpur Medical College Hospital, Rangpur, BGD

**Keywords:** bangladesh, mini-percutaneous nephrolithotomy, renal stones, standard percutaneous nephrolithotomy, stone-free rate, urolithiasis

## Abstract

Background

Renal stone disease constitutes a significant global urological burden, with percutaneous nephrolithotomy (PCNL) serving as the gold-standard treatment for large calculi. Mini-PCNL has emerged to reduce morbidity while preserving efficacy; however, comparative prospective evidence from South Asian settings, including Bangladesh, remains scarce. This study aimed to compare the clinical outcomes, including efficacy and morbidity, of mini-PCNL and standard PCNL in the management of renal stones.

Methods

A prospective comparative study was conducted at the Department of Urology, Chittagong Medical College Hospital, Chattogram, Bangladesh, and two private hospitals in the same region from May 2019 to April 2020. A total of 100 adult patients with renal calculi (1-3 cm) were recruited through nonprobability consecutive sampling and allocated equally into two groups on the basis of surgeon discretion and intraoperative judgment: standard PCNL (24-28 Fr, n = 50) and mini-PCNL (14-20 Fr, n = 50). Operative parameters, postoperative pain (visual analog scale (VAS) score), hemoglobin drop, hospital stay, stone-free rate, renal function, and complications were compared between the groups. Data were analyzed using independent t-tests and chi-square tests, with p < 0.05 considered statistically significant.

Results

Baseline characteristics, including age, sex, and stone size, were comparable between the groups. Mini-PCNL demonstrated a significantly shorter access time (4.28 ± 0.57 vs 7.87 ± 0.39 min, p < 0.001) and fluoroscopy time (3.75 ± 0.51 vs 5.83 ± 0.65 min, p < 0.001) but a longer operative time (94.53 ± 13.56 vs 78.60 ± 17.80 min, p < 0.001). Postoperative pain (VAS score: 5.52 ± 1.24 vs 7.04 ± 1.09, p < 0.001), hemoglobin drop (1.02 ± 0.32 vs 1.31 ± 0.79 g/dL, p = 0.018), and length of hospital stay (2.32 ± 0.46 vs 3.13 ± 0.72 days, p < 0.001) were significantly lower in the mini-PCNL group. Stone-free rates, renal function outcomes, and complication rates were comparable between the two techniques.

Conclusions

In this study, mini-PCNL was associated with lower perioperative morbidity, shorter hospital stay, and reduced fluoroscopy exposure compared with standard PCNL, while stone clearance was comparable between the two groups. However, operative time was longer with mini-PCNL. Given the nonrandomized allocation and potential for selection bias, these findings should be interpreted with caution. Larger randomized multicenter studies are needed to confirm these observations.

## Introduction

Urolithiasis, defined as the formation of calculi within the urinary tract, is a common and clinically significant urological condition worldwide. According to the Global Burden of Disease (GBD) 2021 analysis, approximately 106 million new cases have been reported globally, with a substantial increase of 26.7% between 2000 and 2021 [1]. The prevalence varies widely across regions, ranging from 1% in parts of Asia to as high as 13% in North America, whereas the incidence in Asia, including South Asia, is estimated at 1%-5% [2]. The disease is further characterized by a high recurrence rate, with nearly 50% of patients experiencing recurrence within five years [3]. In addition to its clinical burden, urolithiasis has a considerable economic impact, with healthcare costs projected to increase substantially in the coming years [3]. These factors collectively highlight urolithiasis as an emerging global public health concern.

Percutaneous nephrolithotomy (PCNL) is currently regarded as the gold-standard surgical treatment for large and complex renal stones, particularly those exceeding 2 cm in size. International guidelines, including those from the European Association of Urology and the American Urological Association, strongly recommend PCNL as first-line therapy for such cases owing to its superior stone-free rates compared with alternative modalities such as shockwave lithotripsy and ureteroscopy [4,5]. Standard PCNL typically involves the use of a large-calibre access sheath (24-30 Fr), enabling effective stone fragmentation and retrieval, and has been shown to achieve stone-free rates exceeding 80%-90% [6,7].

Despite its high efficacy, standard PCNL is associated with notable perioperative morbidity, largely attributable to the larger tract size. Bleeding remains the most common complication, with reported transfusion rates ranging from 5% to 18%, whereas overall complication rates may exceed 20% in some series [8]. Additional complications include postoperative pain, infection, and prolonged hospital stays, which may adversely affect patient recovery and healthcare utilization. The association between a larger tract size and an increased risk of bleeding and tissue injury has led to growing interest in techniques aimed at reducing access-related morbidity [9].

Mini-PCNL is generally defined as the use of a smaller access sheath than standard PCNL, typically ranging from 14 to 20 Fr, depending on the technique and available instrumentation [10]. A reduction in tract size significantly decreases the extent of renal parenchymal injury, thereby reducing intraoperative blood loss, postoperative pain, and duration of hospitalization [10,11]. Advances in endoscopic instruments and lithotripsy technologies have further enabled effective stone clearance through smaller tracts, making mini-PCNL a viable alternative to standard PCNL [10].

Several comparative studies and meta-analyses have evaluated the relative efficacy and safety of mini-PCNL and standard PCNL. The evidence suggests that mini-PCNL provides comparable stone-free rates while offering advantages in terms of reduced bleeding, lower transfusion requirements, and shorter hospital stays [11-13]. However, some studies have reported longer operative times and technical challenges associated with mini-PCNL, particularly in patients with greater stone burdens [12]. Despite the growing body of evidence, findings remain heterogeneous, and the applicability of these results across different clinical settings is uncertain.

Importantly, most of the available evidence originates from high-income countries and specialized centers with advanced infrastructure and surgical expertise. In contrast, low- and middle-income countries (LMICs) face unique challenges, including limited access to specialized equipment, variability in surgical expertise, and delayed patient presentation with more advanced disease [14]. The evidence from South Asia remains limited, and existing studies are often retrospective and focused primarily on standard PCNL [15]. As a result, high-quality prospective comparative data evaluating mini-PCNL in these settings are lacking.

Bangladesh lies within a high-prevalence region for urolithiasis, where environmental, dietary, and socioeconomic factors contribute to the disease burden [3]. Patients frequently present at advanced stages due to limited access to healthcare and delayed health-seeking behavior [14]. Although PCNL is increasingly being performed in tertiary centers, the adoption of mini-PCNL remains limited, and comparative evidence between the two techniques in this population is lacking. Therefore, this study aimed to compare the clinical outcomes, including efficacy and morbidity, of mini-PCNL and standard PCNL in the management of renal stones.

## Materials and methods

Study design and setting

A prospective comparative study was conducted in the Department of Urology, Chittagong Medical College Hospital, Chattogram, Bangladesh, along with two selected private hospitals within the same region. The study was carried out over a period of 12 months, from May 2019 to April 2020.

Study population

The study population comprised adult patients diagnosed with renal calculi who were admitted for PCNL during the study period. Patients aged 18 years or older with stone sizes ranging from 1 cm to 3 cm and with preserved renal function were considered eligible. Although renal stones measuring 1-2 cm may be managed with alternative modalities such as shockwave lithotripsy or retrograde intrarenal surgery, the decision to perform PCNL in the included patients was made by the treating urologist on the basis of clinical judgment, stone characteristics, anatomical considerations, anticipated treatment success, and local practice patterns. The diagnosis of renal stones was established through clinical evaluation supported by imaging modalities such as ultrasonography and plain X-ray of the kidney, ureter, and bladder region, with additional intravenous urography where indicated. Patients were excluded if they declined to provide consent, had radiolucent stones, concomitant distal ureteric or lower urinary tract stones, a single kidney, congenital renal anomalies, or anatomical deformities interfering with positioning, such as scoliosis. Additionally, patients with a history of prior renal surgery or PCNL, those requiring multiple punctures during the procedure, and those with uncontrolled diabetes mellitus, chronic kidney disease, bleeding disorders, or active infections were excluded.

Sampling procedure

A nonprobability consecutive sampling technique was employed. Eligible patients were enrolled sequentially after providing written informed consent. The participants were allocated into two groups in a nonrandomized manner on the basis of the operating surgeon’s discretion and intraoperative judgment. Allocation was based on overall procedural suitability, including stone characteristics other than size alone, pelvicalyceal anatomy, access feasibility, and anticipated technical difficulty. Stone size alone was not used as an independent determinant for selecting the surgical technique. Patients anticipated to be technically more challenging or requiring a larger tract size were preferentially managed with standard PCNL, whereas patients considered suitable for smaller tract access were allocated to mini-PCNL. No formal randomization or allocation concealment was performed.

Sample size determination

The sample size was calculated via a standard formula for the comparison of two means, which yielded a minimum required sample size of approximately 96 participants. A total of 100 patients were ultimately included in the study and equally allocated between the two groups, with 50 participants in each arm.

Data collection procedures

Data were collected via a structured data collection sheet designed for the study (Appendix 1). All participants underwent a comprehensive preoperative evaluation, including a detailed history, clinical examination, and relevant laboratory and imaging investigations. These included urine analysis and culture; complete blood count; serum creatinine; coagulation profile; ultrasonography of the kidney, ureter, and bladder; and plain radiography. Additional investigations, such as intravenous urography, were performed when necessary. Patients with positive urine cultures received appropriate antibiotic therapy before surgery, and a sterile urine status was confirmed before intervention. Comorbid conditions such as hypertension and ischemic heart disease were optimized preoperatively, and antiplatelet medications were discontinued at least seven days before the procedure.

All procedures were performed under general anesthesia. Initially, a ureteric catheter was inserted cystoscopically in the lithotomy position. The patient was then repositioned in the prone position, and the pelvicalyceal system was opacified via contrast under fluoroscopic guidance. Percutaneous access was achieved via an 18-gauge needle followed by guidewire placement. Tract dilation was performed via metallic dilators, with tract sizes ranging from 24 to 28 Fr for standard PCNL and 14 to 20 Fr for mini-PCNL. Stone fragmentation was performed via a pneumatic lithotripter, and fragments were retrieved via nephroscopic instruments. At the end of the procedure, a JJ stent was placed, and a nephrostomy tube was inserted.

Postoperatively, stone clearance was assessed via plain radiography on the first postoperative day. Routine postoperative non-contrast computed tomography was not performed in all patients because of resource limitations and local clinical practice patterns. Pain was evaluated via the visual analog scale (VAS) at 24 hours. Hemoglobin levels were measured on the first postoperative day, and complications were recorded. Patients were followed up after three weeks to assess renal function parameters, including serum creatinine and estimated glomerular filtration rate (eGFR).

Measures

Access time was defined as the interval from initial puncture of the collecting system to placement of the Amplatz sheath, whereas fluoroscopy time referred to the total duration of radiation exposure during access creation. The total operative time was measured from the start of cystoscopy to the time of placement of the nephrostomy tube. Stone-free status was defined as the complete absence of calculi or the presence of residual fragments smaller than 5 mm on postoperative plain radiography. Plain radiography was used because routine postoperative non-contrast computed tomography was not available for all patients in the study setting. The length of hospital stay was calculated as the duration from the day of surgery to discharge.

Statistical analysis

The data were entered and analyzed via IBM SPSS Statistics for Windows, Version 23 (Released 2015; IBM Corp., Armonk, NY, USA). Continuous variables are expressed as the means ± standard deviations (SDs), whereas categorical variables are presented as frequencies and percentages. Comparisons between groups were performed via the independent samples t-test for continuous variables and the chi-square test for categorical variables. A p-value of less than 0.05 was considered statistically significant, with a confidence interval set at 95%.

Ethical considerations

Ethical clearance for this study was granted by the Ethical Committee of Chittagong Medical College, Chattogram, Bangladesh (Memo No. CMC/PG/2019/521). Before enrollment, all participants were informed in detail about the purpose and procedures of the study, and written informed consent was obtained from each participant. Participation was voluntary, and the confidentiality and anonymity of all the collected data were strictly maintained throughout the study period. The participants were also given the right to withdraw from the study at any stage without any consequences.

## Results

Table 1 presents the baseline sociodemographic and clinical characteristics of the study participants. A total of 100 patients were included and equally divided into standard PCNL and mini-PCNL groups. The mean age of the participants was 35.18 ± 10.97 years in the standard PCNL group and 39.45 ± 11.14 years in the mini-PCNL group, with no statistically significant difference between the groups (p = 0.06). Male participants predominated in both groups, accounting for 34 (68%) participants in the standard PCNL group and 38 (76%) participants in the mini-PCNL group (p = 0.37). The mean stone size was comparable between the groups (1.74 ± 0.39 cm vs 1.68 ± 0.37 cm, p = 0.43). Similarly, baseline hemoglobin, serum creatinine, and eGFR values were also comparable between the two groups (p > 0.05).

**Table 1:** Baseline sociodemographic and clinical characteristics of the study participants Independent samples t-test was used for continuous variables, and the chi-square test was used for categorical variables. PCNL: percutaneous nephrolithotomy; eGFR: estimated glomerular filtration rate

Variables	Category	Standard PCNL (n = 50)	Mini-PCNL (n = 50)	Test statistic (t/χ²)	p-value
Age (years)	mean ± SD	35.18 ± 10.97	39.45 ± 11.14	1.91	0.06
Sex	Male	34 (68%)	38 (76%)	0.80	0.37
	Female	16 (32%)	12 (24%)		
Stone size (cm)	Mean ± SD	1.74 ± 0.39	1.68 ± 0.37	0.79	0.43
Baseline hemoglobin (g/dL)	Mean ± SD	12.84 ± 1.12	12.91 ± 1.08	t = 0.32	0.75
Baseline creatinine (mg/dL)	Mean ± SD	0.96 ± 0.18	0.94 ± 0.16	t = 0.58	0.56
Baseline eGFR (mL/min/1.73 m²)	Mean ± SD	94.26 ± 12.34	95.18 ± 11.96	t = 0.38	0.71

A comparison of the operative parameters between the two groups is summarized in Table 2. The mean access time was significantly shorter in the mini-PCNL group than in the standard PCNL group (4.28 ± 0.57 minutes vs 7.87 ± 0.39 minutes, p < 0.001). Similarly, the mean fluoroscopy time was significantly lower in the mini-PCNL group (3.75 ± 0.51 minutes) than in the standard PCNL group (5.83 ± 0.65 minutes, p < 0.001). However, the mean operative time was significantly longer in the mini-PCNL group (94.53 ± 13.56 minutes) than in the standard PCNL group (78.60 ± 17.80 minutes) (p < 0.001).

**Table 2:** Comparison of intraoperative parameters between standard PCNL and mini-PCNL Independent samples t-test was used. PCNL: percutaneous nephrolithotomy

Variables	Standard PCNL (n = 50), mean ± SD	Mini-PCNL (n = 50), mean ± SD	t-value	p-value
Access time (minutes)	7.87 ± 0.39	4.28 ± 0.57	36.89	<0.001
Fluoroscopy time (minutes)	5.83 ± 0.65	3.75 ± 0.51	17.64	<0.001
Operative time (minutes)	78.60 ± 17.80	94.53 ± 13.56	5.00	<0.001

Table 3 shows the comparison of clinical outcomes between the two groups. Postoperative pain, assessed via the VAS, was significantly lower in the mini-PCNL group (5.52 ± 1.24) than in the standard PCNL group (7.04 ± 1.09), indicating better postoperative comfort in the mini-PCNL group (p < 0.001). The mean decrease in hemoglobin was also significantly lower in the mini-PCNL group (1.02 ± 0.32 g/dL) than in the standard PCNL group (1.31 ± 0.79 g/dL), reflecting reduced blood loss (p = 0.018). In addition, the mean duration of hospital stay was significantly shorter in the mini-PCNL group (2.32 ± 0.46 days) than in the standard PCNL group (3.13 ± 0.72 days), and this difference was statistically significant (p < 0.001). The stone-free rate was high in both groups, with 96% in the standard PCNL group and 94% in the mini-PCNL group, and the difference was not statistically significant (p = 0.64).

**Table 3:** Comparison of postoperative clinical outcomes between standard PCNL and mini-PCNL Independent samples t-test was used for continuous variables, and the chi-square test was used for categorical variables. PCNL: percutaneous nephrolithotomy; VAS: visual analog scale

Variables	Standard PCNL (n = 50), mean ± SD	Mini-PCNL (n = 50), mean ± SD	Test statistic (t/χ²)	p-value
Postoperative pain (VAS)	7.04 ± 1.09	5.52 ± 1.24	6.48	<0.001
Hemoglobin drop (g/dL)	1.31 ± 0.79	1.02 ± 0.32	2.41	0.018
Hospital stay (days)	3.13 ± 0.72	2.32 ± 0.46	6.64	<0.001
Stone-free rate, n (%)	48 (96.0)	47 (94.0)	0.21	0.64

The changes in renal function parameters are presented in Table 4. The mean change in the serum creatinine level was 0.17 ± 0.04 mg/dL in the standard PCNL group and 0.16 ± 0.06 mg/dL in the mini-PCNL group, with no statistically significant difference between the groups (p = 0.33). Similarly, the mean improvement in eGFR was comparable between the two groups, measuring 10.03 ± 3.04 mL/min/1.73 m² in the standard PCNL group and 9.95 ± 2.44 mL/min/1.73 m² in the mini-PCNL group (p = 0.88).

**Table 4:** Comparison of postoperative renal function changes between standard PCNL and mini-PCNL Independent samples t-test was used. PCNL: percutaneous nephrolithotomy; eGFR: estimated glomerular filtration rate

Variables	Standard PCNL (n = 50), mean ± SD	Mini-PCNL (n = 50), mean ± SD	t-value	p-value
Change in serum creatinine (mg/dL)	0.17 ± 0.04	0.16 ± 0.06	0.98	0.33
Change in eGFR (mL/min/1.73 m²)	10.03 ± 3.04	9.95 ± 2.44	0.15	0.88

Table 5 summarizes the postoperative complications observed in the study. The incidence of fever was slightly greater in the standard PCNL group (10.0%) than in the mini-PCNL group (6.0%), although the difference was not statistically significant (p = 0.45). Sepsis occurred in 2.0% of patients in the standard PCNL group, whereas no cases were reported in the mini-PCNL group (p = 0.31). Blood transfusion was required in 2.0% of patients in the standard PCNL group and none in the mini-PCNL group (p = 0.31). Urine leakage was observed in 2.0% of patients in the standard PCNL group but was absent in the mini-PCNL group (p = 0.31).

**Table 5:** Comparison of postoperative complications between standard PCNL and mini-PCNL Fisher's exact test was done. PCNL: percutaneous nephrolithotomy

Complications	Standard PCNL (n = 50), n (%)	Mini-PCNL (n = 50), n (%)	p-value
Fever	5 (10.0)	3 (6.0)	0.45
Sepsis	1 (2.0)	0 (0.0)	0.31
Blood transfusion	1 (2.0)	0 (0.0)	0.31
Urine leakage	1 (2.0)	0 (0.0)	0.31

## Discussion

The present study demonstrated that mini-PCNL was associated with a reduced operative burden and improved postoperative recovery compared with standard PCNL. Shorter access times, lower fluoroscopy exposure, decreased pain, reduced hemoglobin decline, and shorter hospital stays were observed, whereas stone clearance rates remained comparable. However, the operative duration was longer with the miniaturized approach, and the overall complication rates and renal functional outcomes were similar between the two techniques.

One of the most important observations in the present study was the comparable efficacy of the two techniques, as reflected by the high and statistically equivalent stone-free rates in both groups. This finding is consistent with large-scale evidence demonstrating the noninferiority of mini-PCNL over standard PCNL in the management of renal calculi [13]. A systematic review and meta-analysis by Sharma et al. reported no significant difference in stone-free rates, suggesting that a reduction in tract size does not compromise treatment efficacy [11]. This equivalence may be explained by advances in lithotripsy technology, particularly the use of a holmium:YAG laser, which enables effective fragmentation even through smaller working channels [16]. Improvements in miniaturized nephroscope optics further allow adequate visualization and complete stone clearance. Similar findings have been reported in other comparative studies, suggesting that mini-PCNL maintains comparable effectiveness despite technical modifications [17].

Another notable observation was the difference in operative parameters between the two approaches. Mini-PCNL demonstrated a shorter access time and reduced fluoroscopy exposure, findings that are clinically relevant in minimizing radiation exposure for both patients and surgical teams. The shorter access time may be attributed to simplified dilation techniques that allow more precise tract creation [12]. However, the operative time was significantly longer in the mini-PCNL group, which has been consistently reported in previous studies. The smaller working channel limits rapid fragment extraction, requiring prolonged fragmentation and irrigation [16]. This pattern has also been described in meta-analyses and comparative studies showing a shorter operative duration with standard PCNL [12,17]. These findings indicate that while the efficiency of stone removal may be reduced, the overall effectiveness of mini-PCNL remains unaffected.

The findings from this study suggest that the most clinically relevant advantage of mini-PCNL lies in its reduced morbidity profile. Postoperative pain was significantly lower, and the decrease in hemoglobin was reduced in the mini-PCNL group, indicating decreased renal parenchymal trauma. This is biologically plausible, as a smaller tract size results in reduced tissue disruption and vascular injury [11]. The shorter hospital stay observed in the mini-PCNL group further supports its advantage in terms of postoperative recovery. Similar reductions in hospitalization duration have been reported in large trials and meta-analyses comparing the two techniques [11,13]. In addition, the decrease in hemoglobin aligns with findings from previous studies demonstrating lower blood loss with mini-PCNL [18]. These benefits are particularly important in resource-limited settings such as Bangladesh, where minimizing hospital stay can reduce healthcare burden and improve patient turnover.

The present study also demonstrated comparable safety outcomes between the two procedures. Although complications such as fever, sepsis, and urine leakage were numerically greater in the standard PCNL group, these differences were not statistically significant. This observation is consistent with previous comparative studies reporting similar overall complication rates between the techniques, with some evidence suggesting a trend toward lower complications with mini-PCNL [17]. The absence of transfusion requirements in the mini-PCNL group further supports its safety advantage and is consistent with the literature demonstrating reduced bleeding risk with a smaller tract size [11].

Renal function outcomes were also comparable between the two groups, with both techniques resulting in improvements in the serum creatinine level and the eGFR following stone removal. This finding suggests that the restoration of urinary drainage, rather than tract size, plays a key role in renal functional recovery. Similar findings have been reported in previous studies demonstrating equivalent renal outcomes following both mini-PCNL and standard PCNL [10,19]. These results reinforce that both techniques are effective in preserving renal function, when adequate stone clearance is achieved.

The present study has several strengths. It is one of the few prospective comparative studies evaluating mini-PCNL and standard PCNL in a Bangladeshi setting. It also provides a direct comparison of operative parameters, postoperative outcomes, renal function, and complications in a resource-limited context. However, several limitations should be acknowledged. The relatively small sample size may limit the generalizability of the findings and reduce the statistical power to detect infrequent postoperative complications such as sepsis, transfusion requirement, and urine leakage. Procedures were performed by multiple surgeons, and surgeon-specific experience and technique were not analyzed; therefore, the potential influence of surgeon-related variability on outcomes cannot be excluded. The short follow-up period precluded assessment of long-term outcomes, including stone recurrence and sustained renal function. Most importantly, the nonrandomized allocation based on surgeon discretion and intraoperative judgment may have introduced selection bias, as differences in unmeasured stone complexity or technical difficulty may have influenced both procedure selection and clinical outcomes. Although baseline characteristics, including stone size, were comparable between groups, detailed stone complexity parameters such as stone location, number of stones, hydronephrosis, stone density, access calyx, and validated nephrolithometry scores were not systematically assessed; therefore, residual confounding cannot be excluded. Furthermore, the statistical analysis relied on unadjusted group comparisons, and no multivariable regression analysis was performed to account for potential confounding factors. In addition, subgroup analysis according to stone size categories was not performed. Postoperative complications were recorded according to clinical type but were not prospectively graded using the Clavien-Dindo classification system. Finally, stone-free status was assessed using plain radiography rather than non-contrast computed tomography, which may have reduced the detection of small residual fragments and potentially overestimated the stone-free rate.

## Conclusions

Mini-PCNL was associated with lower postoperative pain, reduced hemoglobin decline, shorter hospital stay, and lower fluoroscopy exposure, while stone-free rates and renal functional outcomes were comparable with standard PCNL. However, operative duration was longer with mini-PCNL. These findings suggest that mini-PCNL may be a useful alternative in selected patients, although the results should be interpreted cautiously due to nonrandomized allocation, potential confounding, and radiographic assessment of stone-free status. Larger randomized, multicenter studies with longer follow-up are needed to confirm these findings.

## Appendices

Appendix 1. Data collection sheet

Participant’s ID no: ..........

A) Demographic information:

a) Name: ..............

b) Age: …….. years

c) Sex: 1 = Male; 2 = Female

d) Religion: 1 = Islam; 2 = Hindu; 3 = Others 

e) Occupation: 1. Service; 2. Business; 3. Farmer; 4. Labourer; 5. Unemployed; 6. Housewife; 7. Student; 8. Others

f) Smoking: 1. Yes; 2. No

g) Address: Vill: ...................; P/S: ...................; Dist: ...............

B) Hospital Reference: ...............; Name of Hospital: ...............

C) Date of Admission: ...............

D) Investigations:

(I) Urine- R/M/E- Pus cells….. /hpf; Epithelial cells …./hpf. RBC…. /hpf.

(II) Hb%- ............... gm/dL

(III) Serum creatinine- ............... mg/dL

(IV) Plain X-ray KUB (100%):

  Side: 1= Right, 2= Left.

  No. of stones: 1. Single, 2.Multiple

  Stone size: …………..mm.

(V) USG of KUB (Comment):

(VI) Intravenous/CT Urogram: Stone location: 1 = Upper calyceal, 2 = Middle calyceal, 3 = Lower calyceal, 4 = Pelvic, 5. Upper ureteric stone.

E) Diagnosis:

F) Treatment plan: 1 = PCNL by standard technique (Group A); 2 = PCNL by mini-percutaneous nephrolithotomy technique (Group B)

G) Puncture location: 1 = Lower calyx, 2 = Middle calyx, 3 = Upper calyx

H) Access time: …………………. seconds

I) Fluoroscopy time for access: ………………. seconds

J) Total Operative time: ……………… minutes

K) Stone clearance by radiology: 1 = Complete, 2 = Residual stone present

L) Per-operative complications: 1 = Excessive bleeding, 2 = Pelvi-calyceal system injury, 3 = Adjacent organ injury.

M) Nephrostomy tube: 1 = Given, 2 = Not Given.

N) Pain: (VNS ………….)

O) Post-operative follow up for complications: 1 = Haematuria, 2 = Fever, 3 = Urinary extravasation, 4 = Urinoma

P) Hb% on 1st POD: ……………… gm/dL

Q) Hospital stay: ………………. days

R) Transfusion required: 1 = Yes, 2 = No

S) Additional procedure advised/performed: 1 = Open surgery, 2 = ESWL, 3 = Re-PCNL, 4 = None

T) Follow up after 3 weeks:

a) Serum Creatinine: …………… mg/dl

b) eGFR: ……………. ml/min

c) Hb%: ……………. gm/dl

____________________________

Name and signature of investigator
